# Trends in breast cancer mortality and analysis of years of life lost among Chinese residents, 2013-2021

**DOI:** 10.3389/fonc.2026.1791685

**Published:** 2026-05-20

**Authors:** Fulei Han, Yue Zhang, Shenghua Wang, Minjun Shen, Xiao Niu

**Affiliations:** 1 Qingdao Maternal & Child Health and Family Planning Service Center, Qingdao, China; 2 Shandong Cancer Hospital and Institute, Shandong First Medical University and Shandong Academy of Medical Sciences, Jinan, China

**Keywords:** AYLL, breast cancer, mortality rate, PYLL, VYLL

## Abstract

**Background:**

This study aims to analyze breast cancer mortality trends and quantify the burden of premature death among Chinese residents from 2013 to 2021 to inform national prevention strategies and resource allocation.

**Methods:**

Data were obtained from the Chinese Cause of Death Monitoring Dataset. Joinpoint regression models were employed to calculate the Annual Percentage Change (APC) and Average Annual Percentage Change (AAPC) to characterize temporal trends. Furthermore, the study utilized Potential Years of Life Lost (PYLL), Average Years of Life Lost (AYLL), and the Value of Years of Life Lost (VYLL) to evaluate the impact of premature mortality on public health and the economy.

**Results:**

From 2013 to 2021, the national age-standardized mortality rate initially rose and then declined, resulting in no statistically significant net change over the study period (AAPC = -0.03, 95% *CI*: -0.56 to 0.51). Rural areas (AAPC = 0.73, 95% *CI*: 0.13 to 1.37) and the Central region (AAPC = 0.86, 95% *CI*: 0.24 to 1.49) exhibited upward trends, contrasting with significant declines in the Eastern region (AAPC = -0.56, 95% *CI*: -1.08 to -0.01). In contrast, the decline in urban areas was not statistically significant (AAPC = -1.06, 95% *CI*: -2.05 to 0.07). Age-specific analysis showed decreased mortality in the 40–44 age group (AAPC = -1.61, 95% *CI*: -3.05 to -0.55) but significant increases in the 65-69 (AAPC = 1.32, 95% *CI*: 0.29 to 2.37) and 70-74 (AAPC = 1.62, 95% *CI*:0.76 to 2.50) cohorts. Furthermore, AYLL and VYLL were disproportionately higher in rural populations and the Western region.

**Conclusions:**

While national breast cancer mortality rates have stabilized, the burden is shifting toward rural regions. The persistence of high premature mortality in resource-limited areas highlights an urgent need for equitable resource allocation and targeted early screening programs to address these widening regional health disparities.

## 1 Introduction

Breast cancer is the most prevalent malignancy among women globally, ranking high in both incidence and mortality and posing a critical threat to public health (1). According to Global Cancer Statistics (GLOBOCAN), there were approximately 2.3 million new breast cancer cases worldwide in 2020, resulting in 685,000 deaths (2). In China, breast cancer has similarly emerged as a major malignancy threatening women’s health, with persistently rising incidence and mortality rates, rendering it an undeniable public health priority.

Previous research has explored the epidemiological characteristics of breast cancer in China. However, many studies exhibit limitations. Some studies cover short time periods or are restricted to specific provinces or cities, making it difficult to capture long-term dynamic trends at the national level (3–7); others fail to incorporate the most recent data, preventing the capture of recent evolutionary characteristics of the disease burden (5). More importantly, traditional mortality indicators cannot fully reflect the complete societal impact of breast cancer, particularly in adequately quantifying the years of life lost due to premature death and their macroeconomic consequences (8). Given that breast cancer diagnoses in China are trending toward younger ages and the population aging process is accelerating (9), relying solely on mortality data is insufficient to support precise health policy decisions and optimal resource allocation.

This study utilizes national mortality surveillance data from 2013 to 2021. We aim to systematically analyze the mortality trends and to examine disparities separately by urban versus rural residence and across Eastern, Central, and Western regions. By employing Potential Years of Life Lost (PYLL), Average Years of Life Lost (AYLL), and Value of Years of Life Lost (VYLL), we quantify the burden of premature death. These findings provide scientific evidence to guide national prevention strategies, optimize resource allocation, and address regional health inequalities.

## 2 Methods

### 2.1 Data sources

Data were obtained from the Chinese Cause of Death Monitoring Dataset. In 2013, the original death registration system of the Chinese Ministry of Health and the national disease monitoring system were integrated and expanded to establish a population death information registration management system (10). This system includes 605 monitoring points covering a population of over 300 million, representing approximately 24.3% of the total Chinese population. This sample size ensures strong national representation. Causes of death were coded according to the International Statistical Classification of Diseases and Related Health Problems, 10th Revision (ICD-10). Breast cancer was identified by code C50. To account for differences in age and gender structures over time, the 2010 Sixth National Population Census was used as the standard population for calculating age-standardized mortality rates.

### 2.2 Statistical analysis

Data management was conducted using Excel 2021 to describe mortality distribution across populations, time, and regions. To analyze trend changes, we employed the Joinpoint Regression Program 4.9.0. We selected the best-fitting model from candidate models with 0 to 4 joinpoints based on the smallest Bayesian information criterion (BIC) (11). In this model, time was used as the independent variable and mortality rate as the dependent variable. We calculated the Annual Percentage Change (APC) and Average Annual Percentage Change (AAPC). An APC or AAPC greater than 0 indicated an upward trend, while values less than 0 indicated a decline. Statistical significance was determined when the 95% Confidence Interval (95% *CI*) did not contain 0 (12).

To assess the dual burden of health and economic loss, we calculated PYLL, AYLL, and VYLL. Based on relevant studies regarding Chinese life expectancy, the threshold for the overall population’s life expectancy was initially set at 80.91 years (13). PYLL quantifies the aggregate life loss from premature death. AYLL reflects the average loss per case. VYLL provides a monetary valuation to measure economic burden. The detailed formulae are described in **Supplementary Text 1**. For the VYLL calculation, we employed the Human Capital Approach. The baseline economic value was derived from the national gross domestic product (GDP) per capita, with 2013 selected as the baseline year. Economic values for subsequent years were adjusted according to actual annual per capita GDP to capture economic growth dynamics (**Supplementary Table 1**). Economic costs were discounted to 2021 present value using a 3% annual discount rate. Sensitivity analyzes employing 0% and 5% discount rates were conducted to assess the stability of economic estimates.

## 3 Results

### 3.1 Mortality trends

The crude and standardized mortality rates for breast cancer among Chinese residents from 2013 to 2021 are detailed in **Table 1**. In 2021, the crude mortality rate was 3.99 per 100,000, and the standardized rate was 3.04 per 100,000.

**Table 1:** Breast cancer mortality rate among Chinese residents from 2013 to 2021.

Year	Crude mortality rate (per 100,000)								Standardized mortality rate (per 100,000)							
	Male	Female	Rural	Urban	East	Central	West	Total	Male	Female	Rural	Urban	East	Central	West	Total
2013	0.17	6.62	2.90	4.31	4.27	2.84	2.62	3.34	0.16	5.80	2.66	3.68	3.54	2.66	2.51	2.99
2014	0.16	6.90	3.04	4.37	4.22	3.11	2.79	3.46	0.15	6.00	2.73	3.81	3.51	2.86	2.65	3.08
2015	0.15	7.45	3.25	4.76	4.49	3.43	3.07	3.74	0.13	6.48	2.93	4.15	3.71	3.15	2.93	3.33
2016	0.15	7.59	3.35	4.71	4.67	3.48	2.97	3.81	0.14	6.50	2.96	4.09	3.83	3.13	2.77	3.33
2017	0.16	7.81	3.50	4.76	4.63	3.71	3.13	3.92	0.15	6.67	3.09	4.12	3.79	3.31	2.95	3.42
2018	0.14	7.76	3.50	4.62	4.66	3.64	3.02	3.88	0.12	6.51	3.05	3.88	3.73	3.19	2.81	3.33
2019	0.12	7.84	3.50	4.72	4.69	3.71	3.03	3.92	0.10	6.19	2.91	3.71	3.57	3.10	2.65	3.19
2020	0.12	7.90	3.59	4.63	4.77	3.75	2.99	3.95	0.10	5.98	2.88	3.46	3.46	3.01	2.55	3.09
2021	0.11	8.00	3.62	4.70	4.76	3.76	3.13	3.99	0.08	5.90	2.84	3.42	3.38	2.93	2.59	3.04

**Figure 1:**
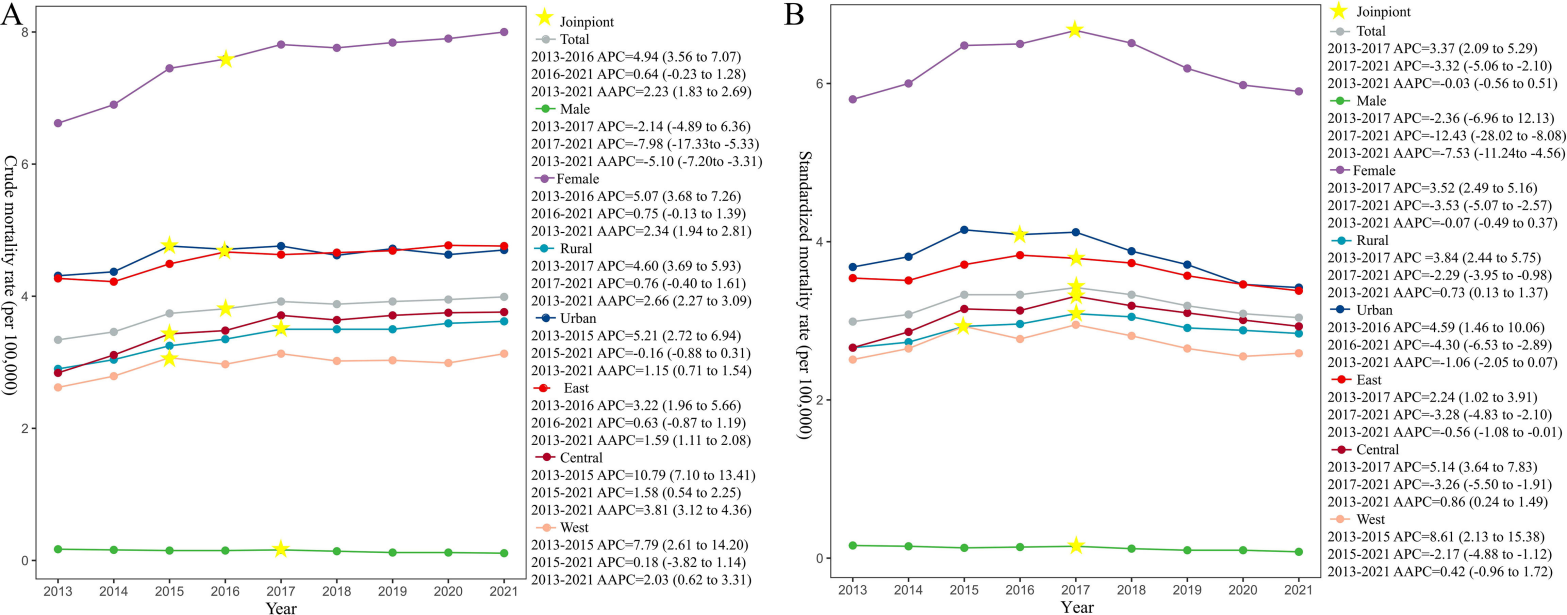
Breast cancer mortality rate among Chinese residents from 2013 to 2021. **(A)** Crude mortality rates. **(B)** Standardized mortality rates.

As shown in **Figure 1**, the national standardized mortality rate initially rose and then declined. Specifically, there was an upward trend from 2013 to 2017 (APC = 3.37, 95% *CI*: 2.09 to 5.29), followed by a decline from 2017 to 2021 (APC = -3.32, 95% *CI*: -5.06 to -2.10). Despite this recent decrease, the overall trend for the entire study period is not statistically significant (AAPC = -0.03, 95% *CI*: -0.56 to 0.51).

Regarding gender, female standardized mortality peaked around 2017. It rose rapidly in the early period (APC = 3.52, 95% *CI*: 2.49 to 5.16) and declined slowly afterward (APC = -3.53, 95% *CI*: -5.07 to -2.57). Overall, female mortality showed a downward trend (AAPC = -0.07, 95% *CI*: -0.49 to 0.37). Meanwhile, male breast cancer mortality showed a consistent downward trend (AAPC = -7.53, 95% *CI*: -11.24 to -4.56).

Trends in urban and rural areas followed a similar pattern. Both saw rapid increases until 2016 or 2017, followed by slow declines. However, over the study period, the standardized mortality rate in rural areas exhibited a general upward trend (AAPC = 0.73, 95% *CI*: 0.13 to 1.37), whereas the downward trend in urban areas was not statistically significant (AAPC = -1.06, 95% *CI*: -2.05 to 0.07). Regionally, the standardized mortality rate in the Central region exhibited an upward trend (AAPC = 0.86, 95% *CI*: 0.24 to 1.49), whereas that in the Eastern region demonstrated a downward trend (AAPC = -0.56, 95% *CI*: -1.08 to -0.01), and the change in the Western region was not statistically significant (AAPC = 0.42, 95% *CI*: -0.96 to 1.72). Notably, the Eastern region saw a rapid rise until 2017 (APC = 2.24, 95% *CI*: 1.02 to 3.91), followed by a plateau. The Central region peaked in 2017 before declining (APC = 5.14, 95% *CI*: 3.64 to 7.83), while the Western region peaked in 2015 (APC = 8.61, 95% *CI*: 2.13 to 15.38).

### 3.2 Age-standardized mortality trends

**Figure 2:**
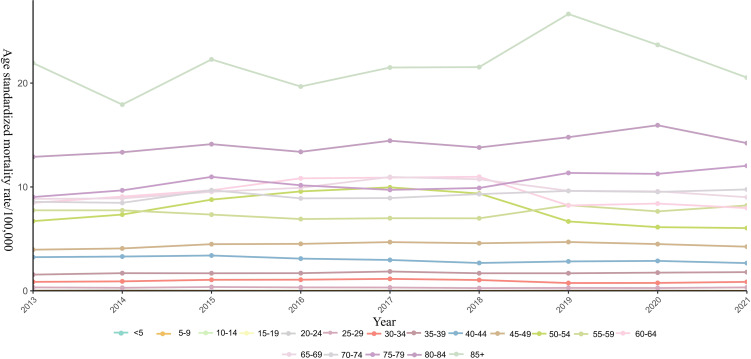
Age-standardized mortality rate of breast cancer among Chinese residents from 2013 to 2021.

Mortality rates increased with age, peaking in the ≥85 age group. Trend analysis reveals that mortality rates generally declined in younger groups. Specifically, the 40–45 age group showed a statistically significant decrease (AAPC = -1.61, 95% *CI*: -3.05 to -0.55). In contrast, older age groups saw increases, with significant upward trends observed in the 65-70 (AAPC = 1.32, 95% *CI*: 0.29 to 2.37) and 70-75 (AAPC = 1.62, 95% *CI*:0.76 to 2.50) cohorts (**Figure 2**; **Supplementary Table 2**).

### 3.3 Analysis of life loss

Between 2013 and 2021, the cumulative PYLL due to breast cancer in the monitored areas was 1,774,752 person years. The AYLL was 25.93 years per death. The total VYLL was 16,093,807,900 USD.

**Figure 3:**
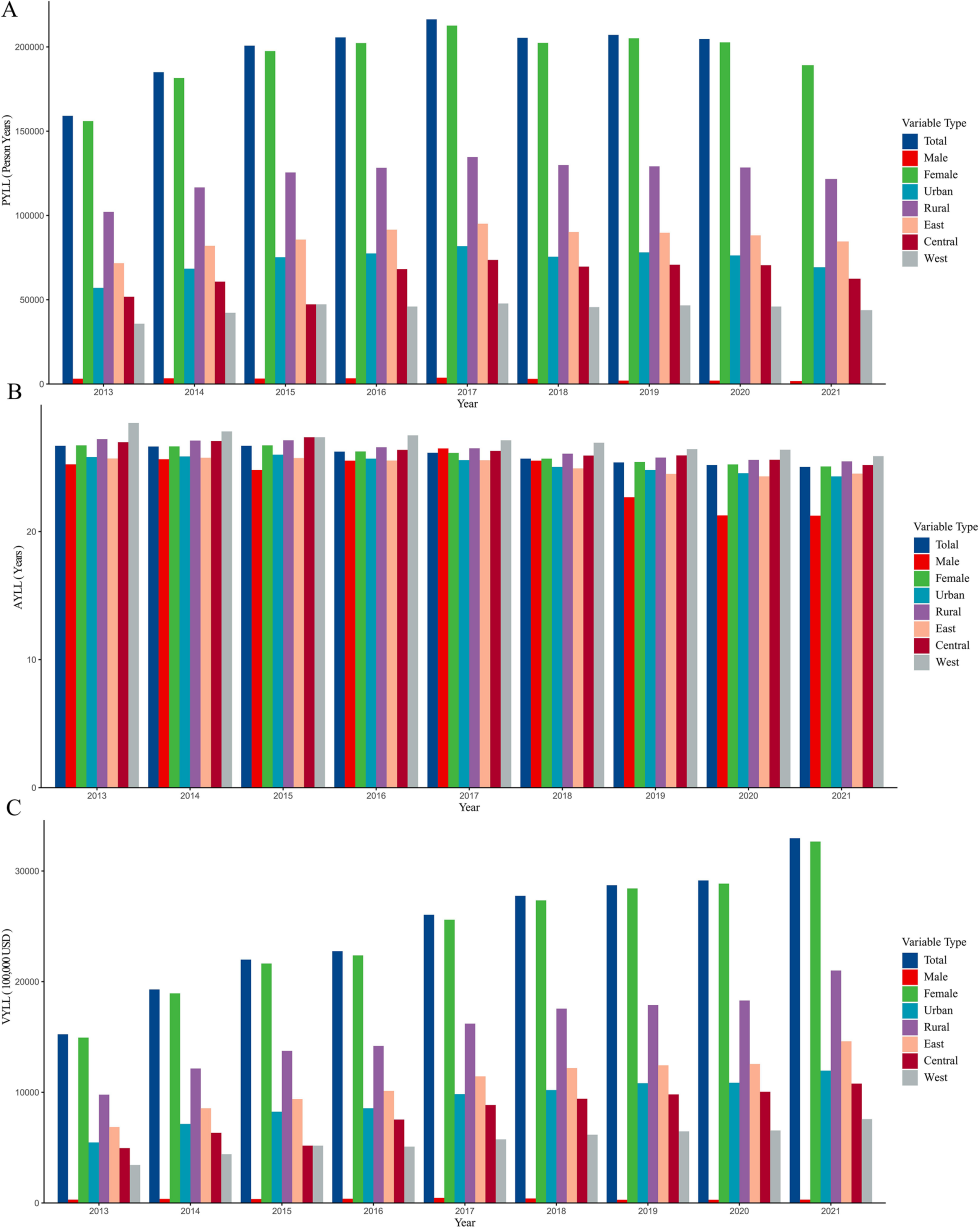
Analysis of potential life loss due to breast cancer among Chinese residents from 2013 to 2021 **(A)** PYLL. **(B)** AYLL. **(C)** VYLL.

Analyzing the distribution of these burdens reveals distinct patterns. As shown in **Figure 3**, AYLL was higher in females than males, higher in rural areas compared to cities, and highest in the Western region compared to Central and Eastern China. Similarly, the VYLL followed the same pattern: higher among females, rural residents, and those in the Western region. This trend remained consistent at discount rates of 0% and 5%, as shown in **Supplementary Figure 1**.

## 4 Discussion

This study utilizes high-quality surveillance data to map the trajectory of breast cancer mortality in China from 2013 to 2021. The findings highlight both the achievements of current control measures and the remaining challenges.

The data indicate a slow but persistent rise in crude mortality during the study period. This trend likely results from the interplay of opposing factors. On one hand, advances in diagnostic technology, the widespread use of targeted and immune therapies, and standardized clinical practices could have improved survival rates (14). These medical advancements may act as a brake on mortality growth. Conversely, China may have witnessed a continuous rise in breast cancer incidence. Based on existing literature, this increase could be linked to greater exposure to risk factors, including changing reproductive patterns, rising obesity rates, and the adoption of sedentary lifestyles (15, 16). Consequently, if these forces are indeed operating, the benefits of improved survival might be diluted by the volume of new patients, potentially contributing to a plateauing or slowly rising mortality rate. However, without directly linked incidence and treatment data, these remain hypotheses that cannot be confirmed within this study.

Gender disparities in mortality trends warrant particular attention. While female breast cancer constitutes the vast majority of cases, male breast cancer mortality demonstrated a consistent and statistically significant decline, substantially outpacing the modest decrease observed in females. This pronounced downward trend in males may reflect lower baseline incidence rates or different biological behaviors of breast cancer in males (17, 18). For females, the initial rise followed by decline after 2017 suggests a transition phase where improved treatment efficacy and early detection programs are beginning to offset rising incidence (19, 20). However, persistent high absolute mortality underscores the continued need for enhanced prevention strategies.

Age-specific analysis reveals contrasting patterns that demand targeted interventions. The significant mortality decline observed in the 40–44 age group suggests that early detection strategies and improved treatment modalities are yielding benefits for younger, premenopausal women. This cohort likely benefits from heightened health awareness, greater screening participation, and more aggressive treatment protocols (21). Conversely, the concerning upward trends in the 65–69 and 70–74 age groups highlight an emerging crisis among postmenopausal elderly populations. These increases likely reflect the confluence of rapid population aging, accumulated exposure to risk factors, potential underdiagnosis, and possibly less aggressive treatment approaches in older patients due to comorbidities or age-related therapeutic considerations (22–24).

There are differences between urban and rural areas, as well as among different regions. Urban areas historically exhibit higher mortality rates, often attributed to higher incidence rates linked to westernized lifestyles (25). However, the gap appears to be narrowing. Urban centers benefit from superior medical resources, established screening systems, and higher health literacy, facilitating early diagnosis and effective treatment (26). In contrast, while rural healthcare capacity has improved, it may lag behind the rapidly rising incidence in these areas (27, 28). The gap in access to standardized care and novel therapeutics means rural mortality rates are not declining as fast as those in cities.

The gradient across Eastern, Central, and Western regions mirrors China’s unbalanced socio-economic development. The prosperous Eastern region bears the heaviest mortality burden due to high baseline incidence, yet it also shows the fastest recent decline in mortality rates—a testament to the efficacy of high-quality medical services (29). Conversely, the Western region faces a “double burden”: a rising incidence due to lifestyle shifts combined with relatively scarce medical resources and lower screening coverage (30). This region represents a critical focal point for future national cancer control strategies.

The life-loss analysis underscores the severe impact of breast cancer on younger demographics. The substantial PYLL indicates that deaths frequently occur among young and middle-aged women, causing significant disruption to social productivity and family stability (31). Compared to diseases predominantly affecting the elderly, the premature nature of breast cancer death is stark (32). This highlights the urgent need to shift the focus of control upstream. Reducing PYLL requires prevention and early detection. Expanding screening coverage, improving screening quality, and promoting health education among young women are the most cost-effective strategies to mitigate this burden (33).

This study has several strengths, including the use of representative, long-term national data and the inclusion of multidimensional life-loss metrics. However, this study has several limitations that warrant consideration. First, we must critically consider ascertainment bias and data quality dynamics. While the Chinese Cause of Death Monitoring Dataset provides extensive national representation, the quality and completeness of death certification can vary spatially and temporally. The observed upward mortality trends in rural, Central, and Western areas may be partially driven by improvements in death registration infrastructure and cancer ascertainment completeness in these developing regions between 2013 and 2021, rather than solely an authentic exacerbation of the disease. Additionally, due to the relatively short study period and the nature of the surveillance data, we were unable to conduct age-period-cohort modeling to disentangle the independent effects of age, period, and birth cohort on breast cancer mortality trends. Finally, owing to the ecological nature of our study design and data availability constraints, we were unable to integrate macro-level ecological indicators to contextualize our mortality findings. Future research should endeavor to link mortality surveillance data with health system indicators and socioeconomic covariates to strengthen the scientific rationale for policy recommendations.

## 5 Conclusions

In conclusion, the burden of breast cancer mortality in China remains substantial, with significant regional and urban-rural disparities persisting. Increasing mortality in rural and central regions, and the overburden of premature deaths in low-resource areas, highlight the urgent need for targeted early detection programs, equitable allocation of resources, and age-specific prevention strategies to reduce the loss of life.

## Data Availability

The original contributions presented in the study are included in the article/**Supplementary Material**. Further inquiries can be directed to the corresponding author.

## Supplementary material

Supplementary Text 1 Formulas for calculating PYLL, AYLL and VYLL.

Supplementary Table 1 Gross domestic product (GDP) per capita.

Supplementary Table 2 The trend of age-standardized mortality rates of breast cancer among Chinese residents from 2013 to 2021.

Supplementary Figure 1 Analysis of VYLL due to breast cancer among Chinese residents from 2013 to 2021 (A)0% discount. (B)5% discount.

## Glossary

APC: Annual Percentage Change
AAPC: Average Annual Percentage Change
PYLL: Potential Years of Life Lost
AYLL: Average Years of Life Lost
VYLL: Value of Years of Life Lost
GLOBOCAN: Global Cancer Statistics
ICD-10: International Statistical Classification of Diseases and Related Health Problems, 10th Revision
BIC: Bayesian information criterion
95% *CI*: 95% Confidence Interval
GDP: Gross Domestic Product
