# Supplementary material for: Trends in breast cancer mortality and analysis of years of life lost among Chinese residents, 2013-2021
Source: Front Oncol. 2026 May 20;16:1791685. doi: 10.3389/fonc.2026.1791685 (PMC13230196; doi:10.3389/fonc.2026.1791685)
Supplement: Supplementary Text 1 — Formulas for calculating PYLL, AYLL and VYLL. [file DataSheet1.pdf]

Supplementary Text 1. Formulas for calculating PYLL, AYLL and VYLL

PYLL quantifies the total years of life lost due to premature mortality before a predetermined standard life expectancy or target age. Compared with crude mortality rate and age-standardized mortality rate, PYLL not only reflects the frequency of death occurrence, but also takes into account the age factor at the time of death, and can better reflect the degree of loss of life years.

$$\text{PYLL}= \Sigma_i N_i \times L_i$$

N_i: Number of deaths in each age- and sex-specific group

L_i: Standard life expectancy at the age of death for the corresponding age group

i: Index representing specific age-sex strata

AYLL represents the mean years of life lost per death, calculated by dividing total PYLL by the number of deaths. It eliminates population size effects and directly measures the "prematureness" of deaths.

$$\text{AYLL}=\frac{\Sigma_i N_i \times L_i}{\Sigma_i N_i}$$

N_i: Number of deaths in each age- and sex-specific group

L_i: Standard life expectancy at the age of death for the corresponding age group

N_i: Number of deaths in each age- and sex-specific group

i: Index representing specific age-sex strata

Economic losses were discounted to present value using the 2021 endpoint as the reference year. We applied a standard 3% annual discount rate to convert the economic burden from 2013 into its 2021 present value. The discounting formula was applied as follows:

$$\text{VYLL}= \Sigma_{i=2013}^{2021} \frac{GDP_{percapita,i} \times N_i \times L_i}{(1+0.03)^{(2021-i)}}$$

N_i: Number of deaths in each age- and sex-specific group

L_i: Standard life expectancy at the age of death for the corresponding age group

N_i: Number of deaths in each age- and sex-specific group

i: Index representing specific age-sex strata

This approach ensures that the economic burden reflects contemporaneous valuations while appropriately weighting earlier years' losses.
