# Supplementary material for: Trends in breast cancer mortality and analysis of years of life lost among Chinese residents, 2013-2021
Source: Front Oncol. 2026 May 20;16:1791685. doi: 10.3389/fonc.2026.1791685 (PMC13230196; doi:10.3389/fonc.2026.1791685)
Supplement: Supplementary Table 1 — Gross domestic product (GDP) per capita. [file DataSheet2.pdf]

Supplementary Table 1. Gross domestic product (GDP) per capita

Year	GDP / USD $
2013	7147
2014	7781
2015	8175
2016	8254
2017	8979
2018	10,085
2019	10,342
2020	10,627
2021	12,887

The data is sourced from the National Bureau of Statistics of China: https://www.stats.gov.cn/sj/ndsj/
