# Supplementary material for: Trends in breast cancer mortality and analysis of years of life lost among Chinese residents, 2013-2021
Source: Front Oncol. 2026 May 20;16:1791685. doi: 10.3389/fonc.2026.1791685 (PMC13230196; doi:10.3389/fonc.2026.1791685)
Supplement: Supplementary Table 2 — The trend of age-standardized mortality rates of breast cancer among Chinese residents from 2013 to 2021. [file DataSheet3.pdf]

Supplementary Table 2. The trend of age standardized mortality rates of breast cancer among Chinese residents from 2013 to 2021

Age group	2013	2014	2015	2016	2017	2018	2019	2020	2021	AAPC
<5	0	0	0	0	0	0	0	0	0	-
5-9	0	0	0	0	0	0	0	0	0	-
10-14	0	0	0	0.01	0	0	0	0	0	-
15-19	0.01	0.01	0.01	0.02	0.01	0	0	0.01	0	-
20-24	0.05	0.08	0.04	0.02	0.03	0.02	0.06	0.05	0.01	-3.74(-14.20 to 4.49)
25-29	0.33	0.29	0.37	0.33	0.32	0.26	0.28	0.27	0.33	-0.31(-5.16 to 4.70)
30-34	0.86	0.91	1.06	1.07	1.15	1.04	0.75	0.76	0.86	-1.01(-3.58 to 1.56)
35-39	1.56	1.70	1.69	1.70	1.86	1.69	1.69	1.75	1.80	1.35(-0.04 to 2.82)
40-44	3.24	3.30	3.40	3.10	2.97	2.68	2.83	2.88	2.67	-1.61(-3.05 to -0.55)
45-49	3.96	4.08	4.49	4.52	4.69	4.58	4.70	4.50	4.25	0.35(-0.85 to 1.81)
50-54	6.71	7.34	8.78	9.57	9.96	9.37	6.68	6.13	6.04	-1.16(-4.05 to 1.47)
55-59	7.75	7.75	7.34	6.91	6.99	6.98	8.24	7.65	8.22	0.43(-0.80 to 1.62)
60-64	8.47	9.07	9.68	10.83	10.90	10.98	8.22	8.40	7.97	-1.14(-4.39 to 1.66)
65-69	8.85	8.90	9.53	9.92	10.96	10.74	9.63	9.58	9.01	1.32(0.29 to 2.37)
70-74	8.57	8.47	9.69	8.90	8.93	9.31	9.62	9.52	9.76	1.62(0.76 to 2.50)
75-79	9.03	9.67	10.97	10.16	9.72	9.90	11.35	11.26	12.04	1.67(-1.73 to 5.49)
80-84	12.90	13.34	14.12	13.38	14.45	13.80	14.79	15.94	14.22	1.46(-0.22 to 3.29)
85+	21.93	17.93	22.28	19.67	21.50	21.54	26.65	23.68	20.53	0.39(-3.01 to 4.05)
