# Supplementary figures and images for: Trends in breast cancer mortality and analysis of years of life lost among Chinese residents, 2013-2021
Source: Front Oncol. 2026 May 20;16:1791685. doi: 10.3389/fonc.2026.1791685 (PMC13230196; doi:10.3389/fonc.2026.1791685)

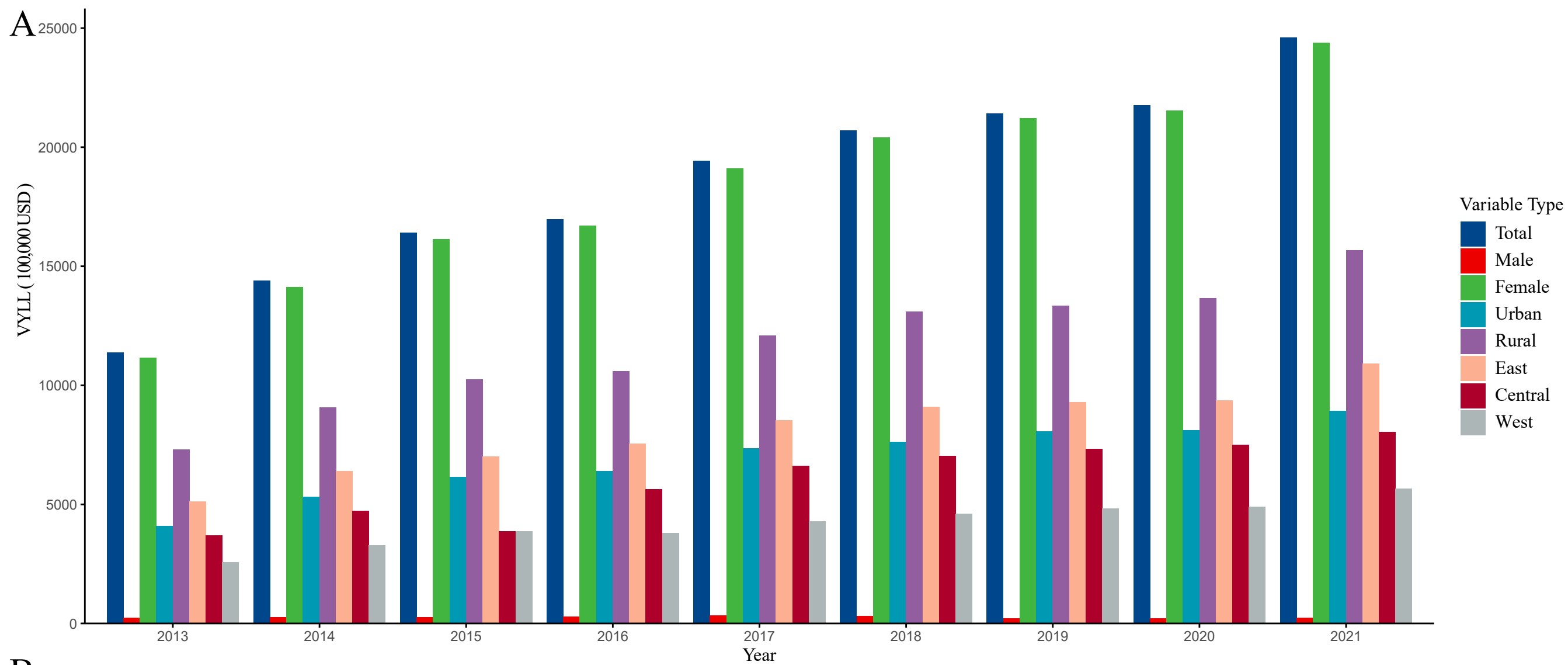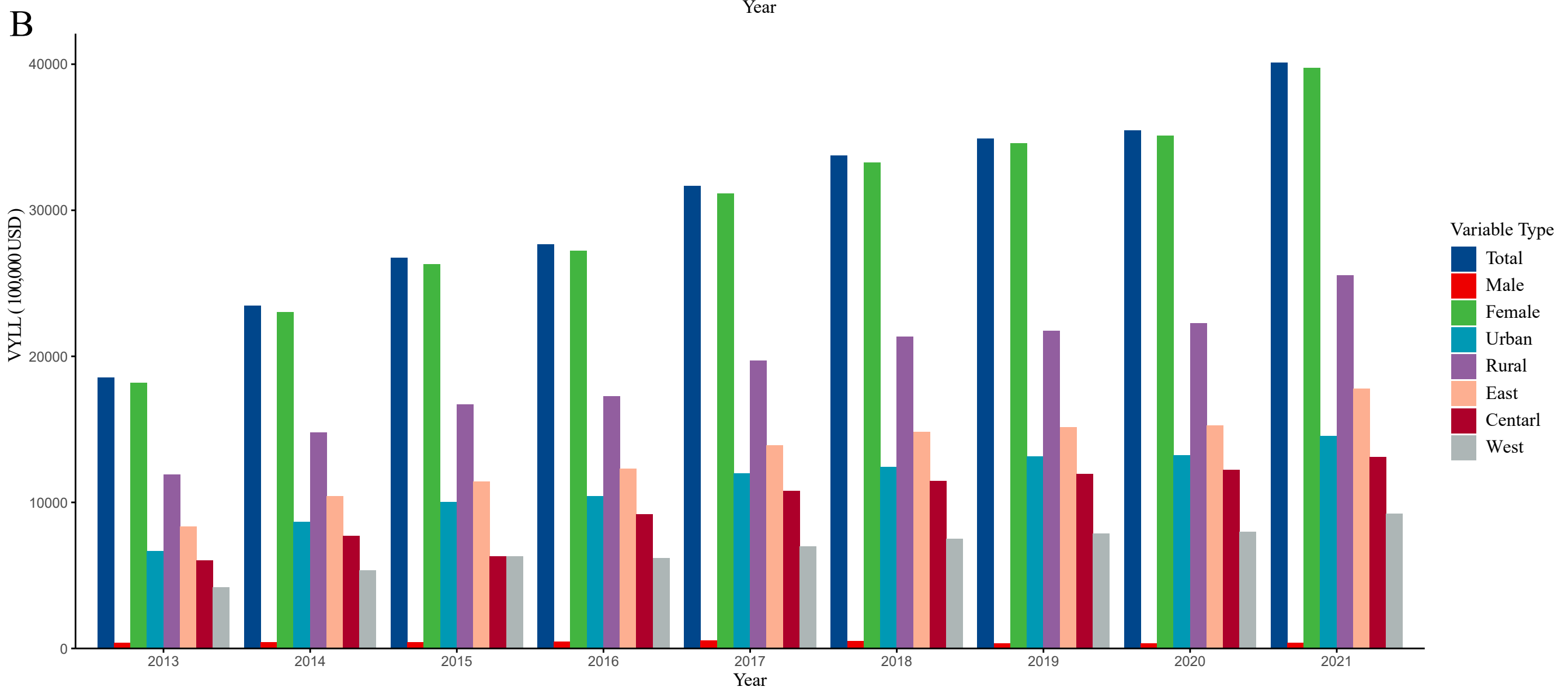

Supplement: Supplementary Figure 1 — Analysis of VYLL due to breast cancer among Chinese residents from 2013 to 2021 (A)0% discount. (B)5% discount. [file DataSheet4.pdf]
